# Salinity stress-induced phosphorylation of INDETERMINATE-DOMAIN 4 (IDD4) by MPK6 regulates plant growth adaptation in *Arabidopsis*

**DOI:** 10.3389/fpls.2023.1265687

**Published:** 2023-10-10

**Authors:** Anamika Rawat, Ronny Völz, Arsheed Sheikh, Kiruthiga G. Mariappan, Soon-Kap Kim, Naganand Rayapuram, Khairiah M. Alwutayd, Louai K. Alidrissi, Moussa Benhamed, Ikram Blilou, Heribert Hirt

**Affiliations:** ^1^Center for Desert Agriculture, Division of Biological and Environmental Sciences and Engineering, King Abdullah University of Science and Technology, Thuwal, Saudi Arabia; ^2^Department of Biology, College of Science, Princess Nourah bint Abdulrahman University, Riyadh, Saudi Arabia; ^3^Institute of Plant Sciences Paris-Saclay IPS2, CNRS, INRA, Université Paris-Sud, Université Evry, Université Paris-Saclay, Orsay, France; ^4^Max F. Perutz Laboratories, University of Vienna, Vienna, Austria

**Keywords:** IDD4, MPK6, salinity stress tolerance, protein phosphorylation, transcription factor, stress signaling

## Abstract

The *INDETERMINATE DOMAIN* (*IDD*) family belongs to a group of plant-specific transcription factors that coordinates plant growth/development and immunity. However, the function and mode of action of IDDs during abiotic stress, such as salt, are poorly understood. We used idd4 transgenic lines and screened them under salt stress to find the involvement of IDD4 in salinity stress tolerance The genetic disruption of *IDD4* increases salt-tolerance, characterized by sustained plant growth, improved Na^+^/K^+^ ratio, and decreased stomatal density/aperture. Yet, *IDD4* overexpressing plants were hypersensitive to salt-stress with an increase in stomatal density and pore size. Transcriptomic and ChIP-seq analyses revealed that IDD4 directly controls an important set of genes involved in abiotic stress/salinity responses. Interestingly, using anti-IDD4-pS73 antibody we discovered that IDD4 is specifically phosphorylated at serine-73 by MPK6 *in vivo* under salinity stress. Analysis of plants expressing the phospho-dead and phospho-mimicking IDD4 versions proved that phosphorylation of IDD4 plays a crucial role in plant transcriptional reprogramming of salt-stress genes. Altogether, we show that salt stress adaption involves MPK6 phosphorylation of IDD4 thereby regulating IDD4 DNA-binding and expression of target genes.

## Introduction

Among the several abiotic stresses, salinity poses a strong concern for plant viability. A high concentration of salt affects plant growth due to hyperionic and hyperosmotic stress. To overcome this, plants have evolved several mechanisms to increase salinity tolerance. One such mechanism operates at the transcriptional level by the activation or repression of genes that can modify plant stress tolerance. Transcription factors (TF) can modulate the different stress response genes and thereby regulate the stress tolerance of plants, e.g. MYBs, WRKYs, NACs *etc*. are some of the TFs that show a strong association with salt-stress responses (Chen et al., 2002; Singh et al., 2002). The IDD family of transcription factors regulate several biological processes in plants (Kumar et al., 2019) and several phosphoproteomic studies have identified IDD transcription factors as targets for phosphorylation (Völz et al., 2019c).

One of the well-studied pathways in response to salinity is the mitogen-activated protein kinase (MAPK or MPK) cascade, which serves in both amplification and transduction of stress signals by phosphorylation through a cascade of three sequentially activated kinase modules: a MAPK kinase kinase, a MAPK kinase and ultimately a MAPK (Ichimura et al., 2002). MAPKs are known to convey besides various biotic, also abiotic stress signals in plants (Sinha et al., 2011). In *Arabidopsis* the MAPKs MPK4 and MPK6 have been shown to be activated by NaCl-treatment (Ichimura et al., 2000). In particular, the MPK6-signaling module was shown to coordinate a plethora of diverse developmental processes, comprising seed, root and stomatal formation, and the regulation of the plant-architecture.

The *INDETERMINATE DOMAIN* (*IDD*)/*BIRD* transcription factor family is plant-specific and highly conserved in monocots and dicots. The IDDs are characterized by a highly conserved N-terminally located ID-domain composed of a nuclear localization signal and four distinct zinc finger domains (ZF1 - ZF4). The 4 ZFs are subdivided into the C_2_H_2_-type ZF1 and ZF2, necessary for DNA-protein interaction, and into the C_2_HC-type ZF3 and ZF4, which are crucial for protein-protein interaction (Kozaki et al., 2004; Colasanti et al., 2006; Hirano et al., 2017).

The maize *ID1* gene was firstly reported as a causal factor for the regulation of floral transition in maize (Colasanti et al., 1998). Since then there have been numerous studies carried out in various plant species showing that *IDD* genes regulate several biological processes in plants, e.g. maintenance of plant architecture and organ development, ammonium metabolism, sugar metabolism, cold stress response, hormone signaling, seed development, secondary cell wall formation and immunity (Kumar et al., 2019; Völz et al., 2019b). *IDD4/IMPERIAL EAGLE* was shown to determine the ad/abaxial leaf development (Reinhart et al., 2013) and organize the ground tissue in roots (Moreno-Risueno et al., 2015). We recently reported that *IDD4* represses the plant-growth and innate immunity against infection by hemibiotrophic and necrotrophic pathogens and is widely expressed during all stages of the rosette leaf development, in trichomes, stomata, epidermal and mesophyll cells (Völz et al., 2019b).

The IDD family members possess a highly conserved putative MAPK binding-motif, indicating post-translational modification upon phosphorylation as the mode-of-action for their mechanistic regulation (Hoehenwarter et al., 2013; Roitinger et al., 2015; Mattei et al., 2016; Völz et al., 2019c). IDD4 harbors a MAPK docking site in between ZF1 and ZF2. Its mode of action depends on its phosphorylation by the immune MAP kinase MPK6 which interacts and phosphorylates IDD4 at serine-73 (S-73) residing in front of the first ZF and at threonine-130 (T-130) located in the second ZF (Völz et al., 2019b). These post-translational modifications determine the IDD4 DNA-binding ability on selected target sequences. Phosphorylated IDD4 exerts a stronger DNA-binding capacity accompanied by the transcriptional induction of the associated gene locus compared to the non-phosphorylated IDD4-variant (Völz et al., 2019b). Chromatin-precipitation studies have revealed the prevalent association of IDD4 to specific cis-regulatory sequences close to the transcriptional start sequence of putative primary target genes (Moreno-Risueno et al., 2015; O'Malley et al., 2016; Völz et al., 2019b; Völz et al., 2019c). These sequences in the 5’ upstream region of target genes contain *ID1*-motifs characterized by the core-sequence AGACAA.

Out of 16 IDD genes found in Arabidopsis, 12 have been shown to be functionally important in various metabolic and development processes, however there are only few studies showing the direct role of IDD genes in abiotic stress response. IDD14 regulates the drought tolerance in plants in an ABA-dependent manner by forming functional complex with ABFs/AREBs (Liu et al., 2022). The cold induced alternative splicing of IDD14 and high temperature induced splice variants of IDD15/SGR5 controls the starch metabolism and shoot gravitropism respectively (Seo et al., 2011; Kim et al., 2016). We previously showed that IDD4 regulates plant immune responses and its activity is controlled by phosphorylation through MPK6 (Völz et al., 2019b), but it’s role in abiotic stress is so far unknown.

Here, we show that *IDD4* acts as a repressor of salt-stress in *Arabidopsis*. Mutations in *IDD4* confer enhanced salt tolerance, sustained plant growth and elevated biomass production. Salt-stress-induced phosphorylation of IDD4 on conserved sites revealed its mechanistic regulation and extends our knowledge of the IDD-TF family in abiotic stress compensation. Our study shows that *IDD4* acts as a hub that links salt stress-induced phosphorylation of IDD4 to the transcriptional regulation of genes involved in salt-stress response thereby tuning - the salt-stress adaptation in *Arabidopsis*.

## Materials and methods

### Plant material and growth conditions

All *Arabidopsis thaliana* lines used in this study are in Columbia-0 (Col.0) background. The *IDD4* transgenic lines used are published (Völz et al., 2019b). The MAPK mutant line used is *mpk6–2* (SALK_073907). Prior to every experiment, seeds were surface sterilized and stratified at 4°C for 2d. The stratified seeds were plated on the ½ strength Murashige and Skoog (MS) medium with 1% agar and plants were grown in plant growth chambers (Percival Scientific) under 16h light: 8h dark condition at 22°C. Unless stated, the plants were grown for 5d on ½ MS and then to apply salt-stress, were transferred onto fresh with ½ MS ± 100mM NaCl (Sigma) and grown for another 16d.

### Bioinformatic analyses/GO term analysis

Sequencing was performed on each library to generate 101-bp paired-end reads on Illumina HiSeq2500 Genome Analyzer platform. Read quality was checked using FastQC and low quality reads were trimmed using the Trimmomatic version 0.32 (http://www.usadellab.org/cms/?page=trimmomatic) with the following parameters: Minimum length of 36 bp; Mean Phred quality score higher than 30; Leading and trailing bases removal with base quality below 3; Sliding window of 4:15. After pre-processing the Illumina reads, the transcript structures were reconstructed using a series of programs, namely, TopHat (ver. 2.1.1; http://tophat.cbcb.umd.edu/) for aligning with the genome, and Cufflinks (ver. 2.2.1; http://cufflinks.cbcb.umd.edu/) for gene structure predictions. For TopHat, the Reference-*Arabidopsis thaliana* (TAIR10) genome (https://www.arabidopsis.org) was used as the reference sequences with a maximum number of mismatches as 2. To identify the differentially expressed genes, the following parameters were used: p-value of 0.05 with a statistical correction using Benjamini Hochberg FDR of 0.05 in cuffdiff. A cut-off of 2 fold up- or down-regulation has been chosen to define the differential expression. After processing the data, visualization of differential expression was done using cummeRbund v2.14.0 (http://bioconductor.org/packages/release/bioc/html/cummeRbund.html).

The AgriGO analysis tool was used to carry out the GO-analysis (http://bioinfo.cau.edu.cn/agriGO/) (Tian et al., 2017) by using significantly differentially expressed genes between the tested conditions (Tian et al., 2017). The protein-network analysis was performed by using STRING (Szklarczyk et al., 2015). The complete *RNA*seq data is available at the GEO repository (GEO accession GSE242075).

### ChIP analyses

Nuclear proteins were extracted from 14 day-old seedlings on half MS medium. After quantification with the Bradford method, equal amounts of proteins were resolved by SDS-PAGE and then transferred to a polyvinylidene difluoride membrane (Bio-Rad) by the use of a Mini-Protean 3 Cell (Bio-Rad). Immunoblot analysis was performed by the use of 1 µg/mL primary polyclonal antibodies raised against GFP (Abcam AB290) and then with secondary antibodies conjugated to alkaline phosphatase. Antibody complexes were detected by chemiluminescence by the use of the Immun-Start AP Substrate kit (Bio-Rad). ChIP assays were performed by the usage of Anti-GFP antibody - ChIP Grade (Abcam) and RNA polymerase II (Santa Cruz) antibodies. Briefly, after plant material fixation in 1% (v/v) formaldehyde, the tissues were homogenized, and the nuclei were isolated and lysed. Cross-linked chromatin was sonicated by the use of a water bath Bioruptor UCD-200 (Diagenode; 15-s on/15-s off pulses, 15 times). The complexes were immunoprecipitated with antibodies overnight at 4°C with gentle shaking and incubated for 1 h at 4°C with 50 μL of Dynabeads Protein A (Invitrogen). Immunoprecipitated DNA was then recovered by the use of the IPure kit (Diagenode) and analyzed by qRT-PCR as described previously by (Volz et al., 2018). An aliquot of untreated sonicated chromatin was processed in parallel and used as the total input DNA control.

The transgenic lines carrying the *IDD4:GFP* construct under the control of the *UBI10* (At4g05320) promoter were used (Völz et al., 2019b). The retrieved binding patterns were normalized by comparison with data obtained from *pUBI10::GFP* expressing plants.

### Salt-stress tolerance assay

The 5d old seedlings with equivalent root length were transferred onto fresh media with ½ MS ± NaCl (Sigma) at the concentrations indicated in figures. The fresh weight of shoots and roots was measured on day 16 after transfer of seedlings. The measured shoots and roots were dried for 2d at 65°C and then used for determining the dry weight. For measuring the leaf area, the leaves of same developmental stage from the plants treated with or without 100mM NaCl for 16d were photographed with Axio Imager 2 (Zeiss) and the leaf area was measured using ImageJ. To determine pavement cell shape, the leaves were mounted on a double sided tape and the upper green tissues were scrapped off. The remaining lower epidermis was imaged using an Axio Imager Z2 microscope (Zeiss) equipped with DIC optics and EC Plan-Neofluar objective. The cell boundaries were traced manually to determine area and perimeter using Imagej. The circularity was calculated as 4π(area)/(perimeter)^2^. The experiment was repeated thrice with 6 plants in each replicate.

### Seed germination assay on salt plates

To quantify effect of salt on seed germination, seeds (grown in same condition and harvested at same time) were surface sterilized and stratified at 4°C for 2d. Then, seeds were plated on ½ MS with 1% agar, supplemented with various concentration of NaCl (as indicated in figure) and placed in plant growth chambers at 22°C with under 16h light: 8h dark photoperiod. The seeds with fully emerged radicle, as observed under a stereo microscope, were considered as germinated and the percentage of germination was scored after 5d of incubation. More than 100 seeds were measured in each replicate and the experiment was repeated thrice.

### Stomatal density and aperture measurement assay

The leaves of plants grown for 16d on ½ MS ± 100mM NaCl were used for the stomatal density and stomatal aperture quantification. The abaxial surface of leaves, after removing the midrib, was stuck on to a double sided tape, already attached to a microscopic glass slide. Then the upper green leaf tissue was scrapped off by scalpel and remaining lower epidermis was imaged using an Axio Imager Z2 microscope (Zeiss) equipped with DIC optics and EC Plan-Neofluar, 40x/0.75 objective for photographing stomatal apertures and 20x/0.75 objective for counting the stomata. For determining the stomatal aperture, the aperture width and total stomatal length of each stoma were manually measured using ImageJ and their ratios were analyzed statistically. A minimum of 30 stomata were measured from each leaf. To determine stomatal density, three random areas from the central region of leaf were photographed and number of stomata per unit area in the microscopic field were counted. The experiment was repeated thrice and samples were prepared from at least 3 leaves from each genotype per biological replicate.

### Measurement of Na^+^ and K^+^ contents

Fresh weight of shoots and roots were measured after 16d of ± 100mM NaCl treatment. The samples were then dried for 2d and digested for another 2d at 65°C by adding 2ml of freshly prepared 1% nitric acid (Sigma Aldrich). The concentrations of sodium and potassium ions in the samples were determined by using a flame photometer (model 425, Sherwood Scientific Ltd., UK).

### MAPK activation and IDD4-GFP immunoprecipitation assay

The *IDD4ox* seedlings grown for 15d on ½ MS agar plates were used for the assay. The seedlings were gently removed from agar plates and incubated in liquid ½ MS for at least 2 hours under the light in growth chamber. Thereafter, 1M NaCl was added to liquid media to reach the final concentration of 150mM. The samples were harvested and frozen in liquid nitrogen at 0, 15 and 30 minutes of salt treatment. Total proteins were extracted by resuspending the homogenized plant tissue in extraction buffer (150mM Tris-Cl pH 7.5, 150mM NaCl, 5mM EDTA, 2mM EGTA, 5% glycerol, 2% PVPP, 10mM DTT, 0.5mM PMSF, 10mM NaF, 10mM Na_2_MoO_4_, 1mM Na_3_VO_4_, 20mM β-glycerophosphate, 10mM SDS and protease inhibitor cocktail from Sigma). For MAP Kinase detection, the total proteins were resolved on 10% SDS-PAGE and analysed by western blot with anti-pTEpY antibody (Cell Signaling) and HRP conjugated anti-rabbit antibody (Sigma). For detection of phosphorylated version of IDD4-GFP the immunoprecipitation (IP) of IDD4-GFP protein was carried out using GFP-Trap agarose beads (ChromoTek). Equal amount of proteins were resolved on 10% SDS-PAGE, and analysed by western blot with anti-IDD4-pS73 antibodies (Abmart) and HRP conjugated anti-rabbit (Sigma). The antigen-antibody complex was detected by ECL Select™ Western Blotting Detection Reagent (GE Healthcare Amersham™) and the ChemiDoc MP imaging system (BioRad). As a loading control for IDD4-GFP protein gel, anti-GFP antibody and for MAP Kinase protein Ponceau S (Sigma-Aldrich) was used.

### Accession numbers

Sequence data for the genes described in this article can be found in TAIR under the following accession numbers: *IDD4* (AT2G02080) and *MPK6* (AT2G43790).

## Results

### *IDD4* coordinates plant growth and salt-stress tolerance in *Arabidopsis*

**Figure 1:**
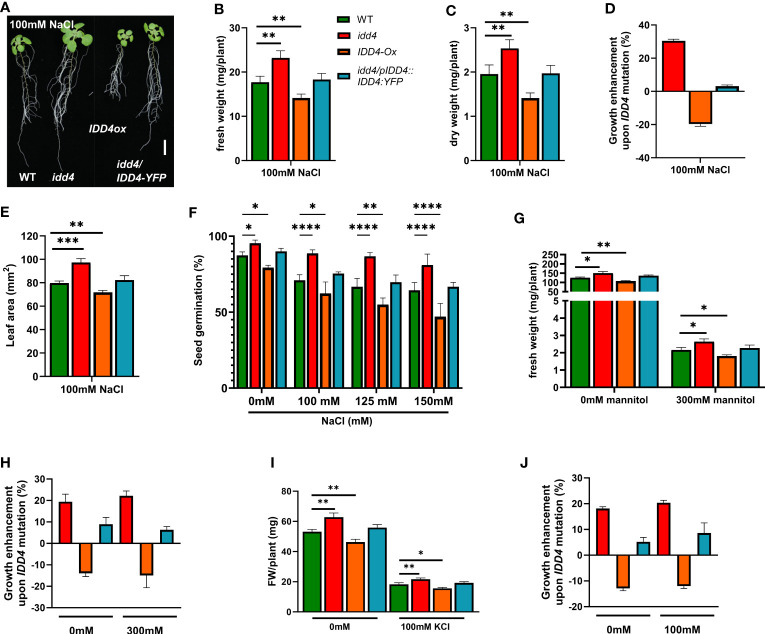
Enhanced salt tolerance of *Arabidopsis idd4* mutant plants. **(A)** Morphology of WT, *idd4*, *IDD4ox* and complementation lines 16d post 100mM salt-stress. Scale bar = 1 cm. **(B)** The fresh weight of plants 16d post 100mM salt treatment. Values represent ± SE (*n* ≥ 25). **(C)** The dry weight of plants 16d post 100mM salt treatment. Values represent average ± SE (*n* ≥ 25). **(D)** The effect of *IDD4* on the growth of *Arabidopsis* under ± 100mM salt-stress condition. **(E)** Leaf area of WT, *idd4*, *IDD4ox* and complementation lines from **(A)**. **(F)** Seed germination assay of WT, *idd4, IDD4ox*, and complementation lines in presence of different concentrations of NaCl. **(G–J)** The fresh weight and percentage in biomass of plants after 16d of ± 300mM mannitol **(G, H)** and ± 100mM KCl **(I, J)** treatment respectively. Values represent average ± SE, (*n* ≥ 300). Significant differences from WT under respective condition is indicated by *. Each bar in the graphs represents an average of three biological replicates ± SE; * *p* ≤ 0.05, ** *p* ≤ 0.01, *** *p* ≤ 0.001, **** p < 0.0001 [Student’s *t* test for **(B, C, E, G, H)**; Two way ANOVA with Dunnett’s multiple comparison for **(F)**].

In order to understand the role of IDD4 in abiotic stress responses, we examined the *idd4* mutant, *IDD4* overexpressor *(IDD4ox)*, *IDD4* complementation lines (*idd4*/*IDD4::IDD4-YFP*), and WT plants on ½ MS agar plates supplemented with 100mM NaCl. After 16d of salt treatment, *idd4* plants displayed enhanced growth in both shoot and root systems, while *IDD4ox* were significantly more sensitive to salt treatment than *idd4* or WT plants (**Figure 1A**). The growth of the complementation line was indistinguishable from WT, indicating that the growth-promoting effect of *idd4* can be traced back to the T-DNA insertion in its open-reading frame.

The effect of salt was evaluated in more detail by measuring the fresh and dry weight of plants grown for 16d on ½ MS ± 100mM NaCl and under non-salt conditions. Without salt-treatment, the *idd4* mutant showed a slight increase in fresh and dry weight, whereas the growth of the *IDD4ox* lines were found slightly behind that of WT (**Supplementary Figures 1A–D**). However, salt-stress conditions strongly exacerbated the tendencies observed in *idd4* and I*DD4ox* under non-salt conditions. The *idd4* mutant plants showed strong growth, with an enhanced salt-stress compensation, while the overexpression of *IDD4* resulted in a dwarfed, salt sensitive plants, with strongly reduced in fresh and dry weight (**Figures 1B-D**). In order to verify whether the phenotype we observed is salt specific or is a general response to other stresses, we treated the *idd4*, *IDD4ox* on ½ MS plates supplemented with mannitol or KCl. Interestingly, unlike the salt treated plants, we did not observe a significant change in the growth enhancement of plants that were given either mannitol or KCl stress as compared to controlled plants (**Figures 1G-J**). These results suggest *IDD4* as a plant-growth regulator under salt-stress conditions.

To gain further insight into the possible role of *IDD4* in coordinating growth and stress tolerance in *Arabidopsis*, we measured the area of true leaves after 16d of salt-stress. When compared to WT, the leaf surface area in *idd4* plants was significantly increased, while that of *IDD4ox* showed a slight reduction (**Figure 1E**; **Supplementary Figures 1E, F**).

The increased leaf area might be one of the possible reasons that *idd4* have bigger plants and increased biomass. One of the factors that govern the final organ size is cell expansion. To analyze the extent to which the cell size contributes to enlarged leaves in *idd4*, we quantified the pavement cell (PC) shape parameters and observed significant differences between the area and circularity of PC in *idd4* mutants. The *idd4* mutant displayed an increase in PC area, while the mean PC circularity was decreased under salt as well as non-salt conditions (**Table 1**; **Supplementary Figure 1G**), showing the increase in cell shape complexity. The PC area and circularity in *IDD4ox* were similar to WT except for the slight decrease in cell area under non-salt conditions (**Table 1**). Circularity is a descriptor of cell shape complexity and a decrease in circularity indicates increased PC lobes. The reduction in mean circularity and increase in the area of *idd4* mutants could be due to an increase in the cell lobe number of PC, resulting in increased cell area and thereby the bigger leaves in these plants.

**Table 1:** Quantitative analysis of pavement cell shape phenotype of WT, *idd4* and *IDD4ox*.

	Area (µm^2^)		Circularity^#^	
Genotype	0mM NaCl	100mM NaCl	0mM NaCl	100mM NaCl
WT	3708.3 ± 151.6 (a)	2659.7 ± 118.4 (c)	0.28 ± 0.01 (a)	0.34 ± 0.01 (c)
*idd4*	5805.3 ± 297.3 (b)	3709.7 ± 142.1 (a)	0.20 ± 0.01 (b)	0.29 ± 0.01 (a)
*IDD4ox*	2643.2 ± 125.4 (c)	2519.6 ± 107.6 (c)	0.28 ± 0.01 (a)	0.39 ± 0.01 (d)

^#^Circularity is calculated as 4π(area)/(perimeter)^2^.

Values represent mean ± SE of three biological replicates. Different letters in parentheses denote significant difference as calculated using Two-Way ANOVA, Tukey’s HSD, p ≤ 0.05, n = 120 (non-salt samples) and 180 (salt samples).

To further elucidate the salt-stress response of *idd4 and IDD4ox* lines, we assessed the ability of these lines to germinate on media supplemented with different concentrations of salt. Under non-salt conditions, *idd4* seeds germinated at slightly higher (9%) and *IDD4ox* lines at significantly lower rates (8%) than WT (**Figure 1F**). Under increasing salt concentrations, these tendencies between the *idd4, IDD4ox*, and WT lines were strongly enhanced (**Figure 1F**). In contrast, no significant differences from WT were observed for the *IDD4* complementation line (**Figure 1F**). Taken together, these results suggest the role of *IDD4* in coordinating plant growth and salt-stress tolerance in *Arabidopsis*.

### IDD4 controls both stomatal density and opening in response to salt-stress

**Figure 2:**
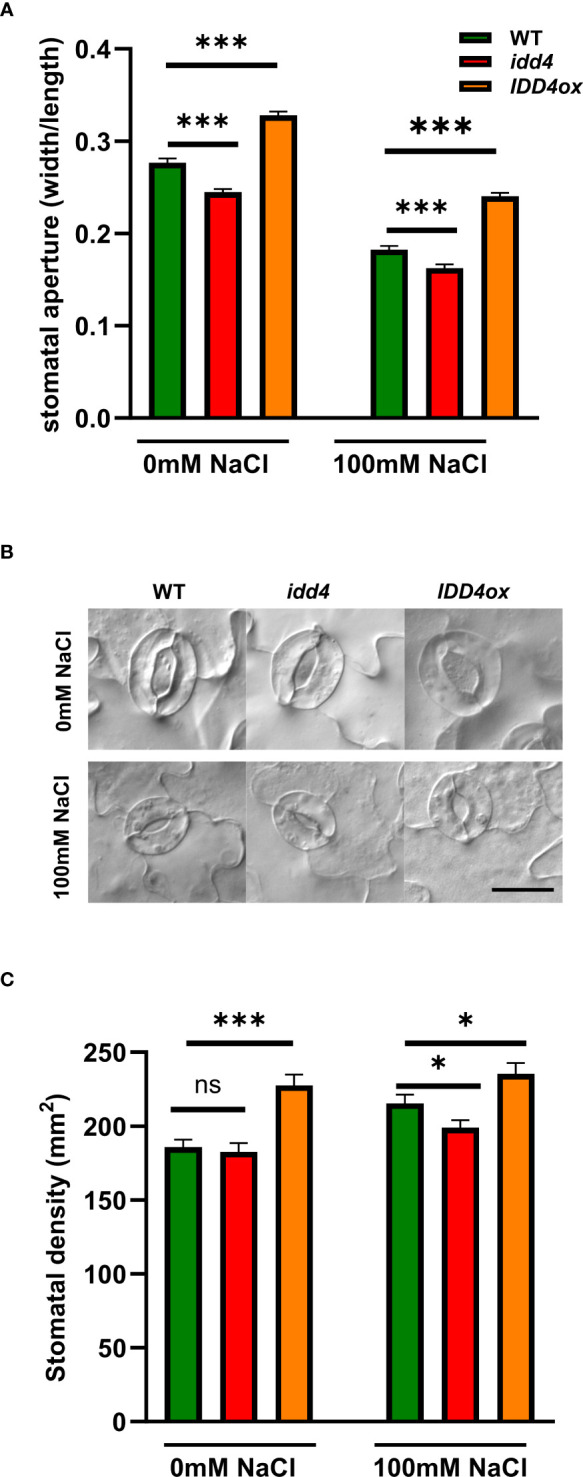
*IDD4* regulates stomatal patterning and aperture in response to salt-stress. **(A)** Size of stomatal aperture in WT, *idd4* and *IDD4ox* after 16d of ± 100mM salt treatment. Each bar represents an average of three biological replicates ± SE, *n* = 405. **(B)** Representative image of stomata of WT, *idd4* and *IDD4ox* after 16d of ± 100mM salt treatment. Scale bar = 20 μm. **(C)** Stomatal density in WT, *idd4* and *IDD4ox* after 16d of ± 100mM salt treatment. Each bar represents an average of three biological replicates ± SE, *n* = 20. * *p* ≤ 0.05, *** *p* ≤ 0.001 (Student’s t-test), *ns* = non significant.

One of the key features of plants under short term salinity stress is the regulation of the stomatal opening, thereby preventing excessive water loss and efficiently coordinating water availability with growth (Munns and Tester, 2008). To evaluate whether the maintenance of growth of *idd4* plants under salt-stress is related to decreased water loss due to a reduction in stomatal transpiration, we decided to look into stomata of stressed and non-stressed plants. Under non-salt conditions, *idd4* showed decreased stomatal aperture when compared to WT (**Figures 2A, B**). In contrast, *IDD4ox* plants showed significantly more open pores. The stomatal pore size showed a significant reduction under the salt-stress, with *idd4* having predominately closed while *IDD4ox* significantly open stomata when compared to WT (**Figures 2A, B**).

Another level of plant adaptation to long term stress conditions is to adjust stomatal development to determine the number of stomata per leaf area, i.e. stomatal density. Under non-salt conditions, *idd4* did not show any differences in stomatal density when compared to WT (**Figure 2C**). In contrast, *IDD4ox* plants showed strongly increased levels of stomatal density. Under salt-stress, while stomatal density was decreased in *idd4*, *IDD4ox* plants showed increased numbers when compared to WT (**Figure 2C**), so that *IDD4ox* always had increased stomatal density and remained more open than WT or *idd4* which both showed the opposite tendencies (**Figure 2**). These results suggest that *IDD4* is involved in regulating stomatal density under salt stress conditions. In summary, these data indicate that *IDD4* plays an important role in regulating stomatal density and physiology in response to environmental signals such as salt-stress.

### *IDD4* controls shoot sodium and potassium ion contents under salt-stress

**Figure 3:**
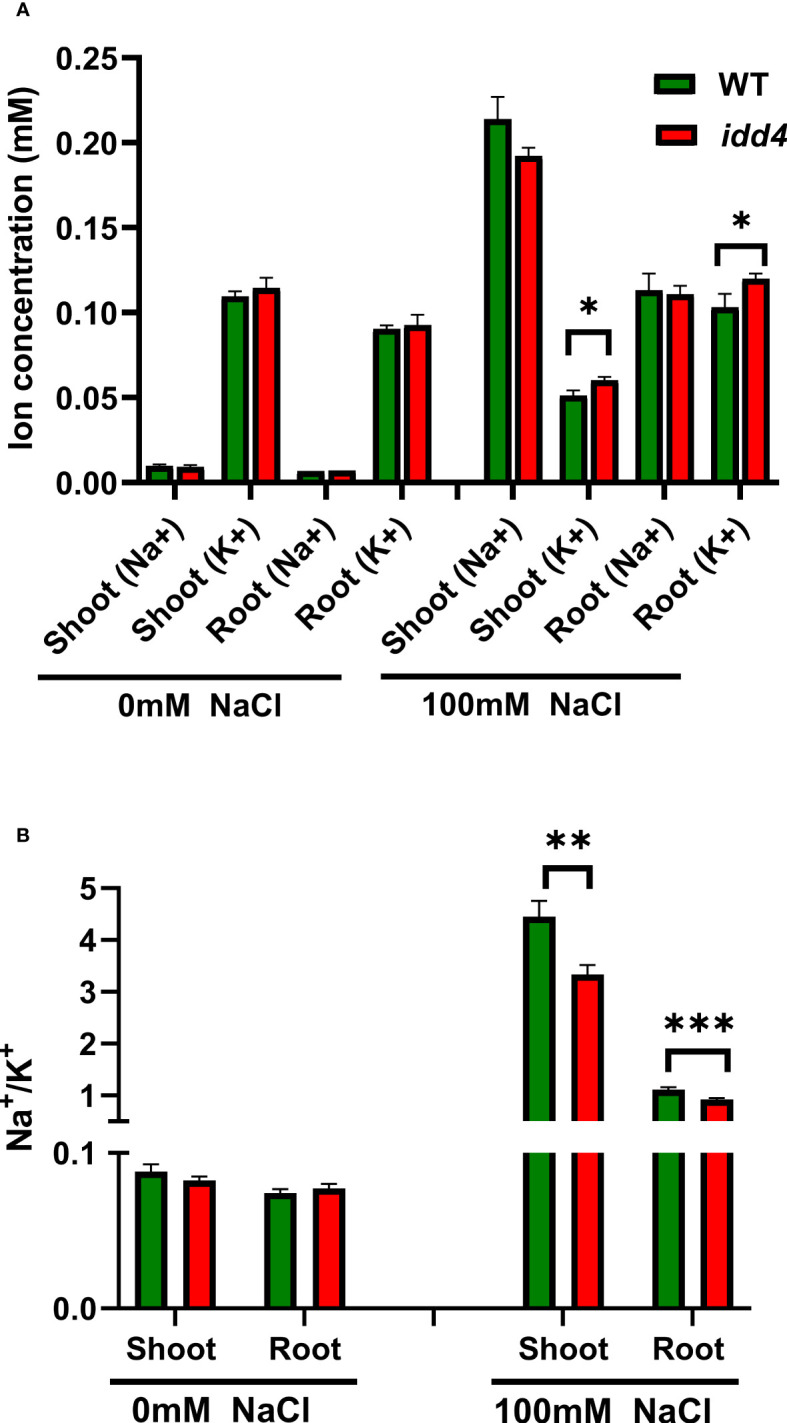
*IDD4* affects the Na^+^/K^+^ ratio under salt-stress conditions. **(A)** Na^+^ and K^+^ ion concentrations in WT and *idd4* treated with ± 100mM NaCl for 16d. Each bar represents an average of three biological replicates ± SE (*n* ≥ 25). **(B)** Na^+^/K^+^ ratios in shoot and root of WT and *idd4* from **(A)**. Values represent average ± SE. Significant differences from WT under respective condition is indicated by * *p* ≤ 0.05, ** *p* ≤ 0.01, *** *p* ≤ 0.001 (Student’s *t* test).

The salinity tolerance of *Arabidopsis* depends on the Na^+^/K^+^ ratio, especially by increasing K^+^ contents (Sun et al., 2015). To investigate whether the underlying salinity tolerance of *idd4* is conferred by changes in the Na^+^/K^+^ ratio, we measured the Na^+^ and K^+^ contents in shoots and roots of salt-treated and non-treated WT and *idd4* plants. Under non-saline conditions, there was no difference in the sodium and potassium ion contents of WT and *idd4* plants, and the Na^+^/K^+^ ratio remained similar in both shoots and roots (**Figures 3A, B**). However, under salt conditions, *idd4* plants not only accumulated fewer sodium ions in shoots but the amount of potassium ions was also found to be significantly higher in both shoots and roots (**Figure 3A**). These changes in ion contents were reflected in the lower Na^+^/K^+^ ratios of *idd4* in both shoots and roots (**Figure 3B**). These results suggest that *IDD4* influences salt-stress adaptation by regulating the Na^+ ^and K^+^ contents of *Arabidopsis* shoots and roots.

### Salinity-induced phosphorylation of IDD4 by MPK6

In our previous study, we showed that the DNA-binding ability of IDD4 depends on its phosphorylation status and that IDD4 is specifically phosphorylated by MPK6 upon pathogen infection (Völz et al., 2019b). To investigate whether MPK6 and IDD4 respond to salt-stress, we examined their phosphorylation upon salt treatment in a time-dependent manner in GFP-tagged *IDD4ox* lines.

**Figure 4:**
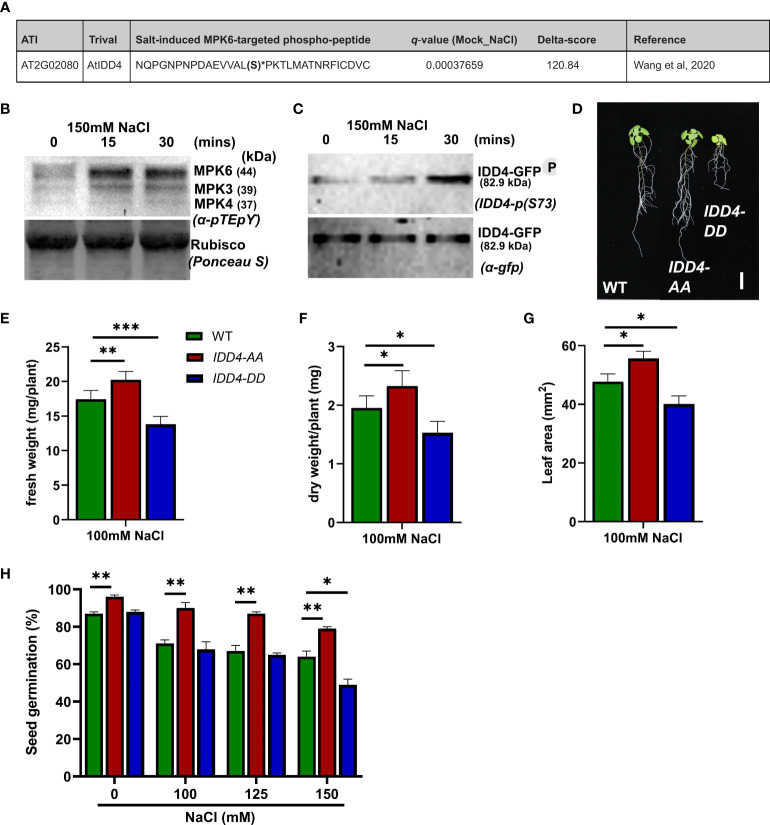
*Arabidopsis IDD4* phospho-dead and phospho-mimic mutants show opposite responses towards salt-stress. **(A)** IDD4 phospho-peptide identified as NaCl-induced heavy 18O-phosphorylation site from MPK6 *in vitro* kinase reaction (Wang et al., 2020). **(B)** Salt-induced MAP kinase activation in *IDD4ox* lines. **(C)** The phosphorylation of IDD4 in *IDD4ox* lines after salt treatment at various time points. **(D)** Morphology of WT, *IDD4-AA and IDD4-DD* lines after 16d of 100mM salt-stress. Scale bar = 1 cm. **(E)** The fresh weight of plants after 16d of 100mM salt treatment. Values represent average ± SE (*n* ≥ 25). **(F)** The dry weight of plants after 16d of 100mM salt treatment. Values represent average ± SE (*n* ≥ 25). **(G)** The leaf area of WT, *IDD4-AA and IDD4-DD* lines from **(C)**. **(H)** Seed germination assay of WT, *IDD4-AA and IDD4-DD* lines on ½MS ± various NaCl concentration. Values represent average ± SE, (*n* ≥ 300). Significant differences from WT under respective condition is indicated by *. Each bar in the graphs represents an average of three biological replicates ± SE; * *p* ≤ 0.05, ** *p* ≤ 0.01, *** *p* ≤ 0.001 (Student’s *t* test).

The activation of MAP kinases depends on the phosphorylation of the TEY motif in the activation loop that can be determined by Western blotting of total protein extracts with a pTEpY antibody. We found that the endogenous MAP kinases MPK6 and MPK3 were phosphorylated at the TEY motif and consequently activated at 15 and 30 min after salt treatment (**Figure 4B**). These findings are in accordance with previous results showing the salt-induced phosphorylation of MPK6 (Ichimura et al., 2000; Teige et al., 2004; Liu et al., 2015).

In a recent phospho-proteomic study following salt-application, isotope-labeled phosphorylation of enriched *in-vivo* phosphorylated peptides (Wang et al., 2020) revealed IDD4 as the substrate of the activated MAP kinase MPK6 (**Figure 4A**). To evaluate the salt-induced S-73 IDD4 phosphorylation (Völz et al., 2019b), we generated an IDD4-pS73 antibody, which specifically detects the MPK6-targeted phosphorylation site of IDD4 (**Supplementary Figure 2**). Using a transgenic line expressing a GFP-tagged version of IDD4 (*IDD4ox* lines) (Völz et al., 2019b), we detected the IDD4 phosphorylation by Western blotting using the phospho-specific IDD4 antibody. We observed a strong increase in the signal intensity after 30 min following salt treatment (**Figure 4C**) thereby confirming the results of the phospho-proteomic study carried out previously (Wang et al., 2020). Western blotting with a GFP antibody confirmed that equal amounts of IDD4-GFP were immunoprecipitated from the crude protein extracts (**Figure 4C**). Altogether, our results indicate S-73 phosphorylation of IDD4 after salt-application and suggest MPK6-as an upstream regulator of salt-dependent IDD4 phosphorylation.

### Phosphorylation of IDD4 determines its ability to confer salt-stress tolerance

To unravel the biological role of IDD4 phosphorylation to salt-stress tolerance, we analyzed the contribution of phospho-dead and phospho-mimic versions of *IDD4* under saline conditions. We used transgenic lines that expressed alanine (A) or aspartate (D) substituted IDD4 corresponding to the previously identified MPK6-targeted phosphosites S-73 and T-130 (Völz et al., 2019b). As shown in **Figure 4D** and **Supplementary Figure 3A**, phospho-dead *IDD4-AA and* phospho-mimicking *IDD4-DD* lines showed different behavior to WT after 16 days on 100mM salt media. Whereas *IDD4-AA* plants showed increased, *IDD4-DD* showed decreased biomass in terms of fresh weight and dry weight, when compared to WT plants (**Figures 4E, F**; **Supplementary Figures 3B, C**). The changes in the shoot size of *IDD4* transgenic lines were also reflected in their leaf areas, where *IDD4-AA* showed increased and *IDD4-DD* decreased levels (**Figure 4G**; **Supplementary Figure 3D**). These results indicate that the phosphorylation status of IDD4 determines the adaptability of *Arabidopsis* to salt-stress. Finally, when the seed germination efficiency of these transgenic lines was determined under salt-stress, *IDD4-AA* showed improved germination rates than WT (**Figure 4H**). In contrast, at all salt concentrations, *IDD4-DD* displayed impaired germination rates when compared to WT or *IDD4-AA* plants (**Figure 4H**). These data show that *IDD4-AA* behaves similarly to *idd4* while *IDD4-DD* responds to salt-stress like the *IDD4ox* line. These outcomes revealed that IDD4’s mode of action depends on its phosphorylation status which is vital for plant salt-stress adaption.

### The knock-out of *IDD4 in mpk6* promotes plants growth under salinity stress

IDD4 interacts with and is a substrate for phosphorylation by MPK6 (Völz et al., 2019b). To gain further insight into the role of MPK6 and IDD4 in salinity stress, we generated the *mpk6/idd4* double mutant and analyzed the biomass and growth under mock and salinity stress.

**Figure 5:**
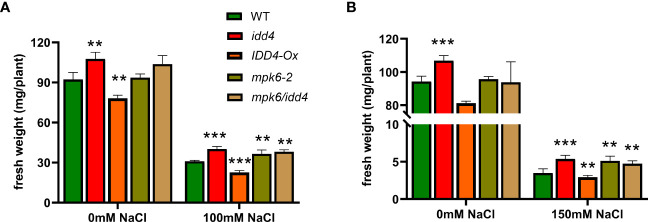
Phenotype of WT, *mpk6-2 and mpk6/idd4 double* mutants under salt-stress. **(A)** The fresh weight of plants after 16d of ±100mM salt treatment. **(B)** The fresh weight of plants after 16d of ±150mM salt treatment. Each bar in the graphs represents an average of three biological replicates ± SE, (*n* ≥ 18), ** *p* ≤ 0.01, *** *p* ≤ 0.001 (Student’s *t* test).

We found, in accordance with the *idd4* and *mpk6-2* single mutants, that the *mpk6/idd4* double mutant was able to withstand salt stress better compared to WT, while no such effect was seen under non-salt conditions (**Figure 5A**).

We further tested the lines under increased salt conditions (150mM NaCl), and found a similar trend of increased tolerance towards salinity in *idd4*, *mpk6-2* and *mpk6/idd4*, while *IDD4-Ox* proved to be hypersensitive towards salinity stress (**Figure 5B**).

These results unravel that IDD4 along with its upstream regulator MPK6 act as repressors of salt-stress adaption.

### *IDD4* acts as a repressor of salt-stress response

In order to analyze how *IDD4* affects the transcriptome following salt-stress we performed *RNA*-seq analysis on 3 biological replicates of 14 day-old *idd4 and* WT seedlings without and after salt treatment (100mM NaCl). A close to linear correlation coefficient of WT and *idd4* (0.988), WT and *idd4* (NaCl) (0.989), were obtained when taking into account the expression profiles for all transcripts. The strict correlation with a value of almost 1 indicates that a loss-of-function of *IDD4* does not interfere with general gene expression but instead influences subsets of genes in particular biological processes. At a stringency of *p*<0.05 in salt-treated *idd4* mutants, 248 differentially expressed genes (DEGs) (**Supplementary Data 1**) were identified that show a log_2_-fold change from 0.66 to 3.11 of positively regulated genes and from -2.11 to -0.63 of negatively regulated genes, among which 174 genes are up- and 74 genes are down-regulated.

**Figure 6:**
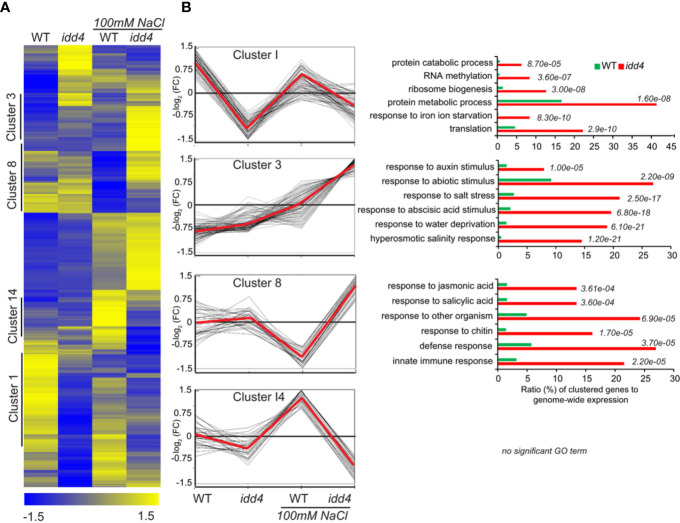
Differential expression of Arabidopsis *idd4* mutant plants in response to salt-stress **(A)** Hierarchical clustering of WT and *idd4* transcriptome with and without salt treatment. The original FPKM values were adjusted by normalized genes/rows and subsequently processed by hierarchical clustering using average linkage method using MeV4.0. Blue and yellow color indicates relatively low and high expression levels, respectively. For the complete gene list see **Supplementary Data 1**. **(B)** Centroid graph (red) and individual expression graphs of differentially regulated genes dedicated to clusters 1, 3, 8, and 14. Besides the graph chats, gene ontology annotations are presented for cluster 1, 3 and 8. GO terms were determined by using the agriGo database (TAIR10).

Hierarchical clustering of significantly deregulated genes (*p*<0.05) in *idd4* before and after NaCl treatment, by using normalized FPKM values, revealed distinct differences in gene expression patterns in *idd4* after NaCl perception (**Figure 6A**; **Supplementary Data 2**).

To categorize DEGs into functional modules, gene ontology (GO) enrichments were determined using AgriGO platform (Tian et al., 2017) (TAIR10). The oppositely regulated genes between WT and *idd4* without salt-stress and after salt-perception are shown in Cluster I (**Figure 6B**; **Supplementary Data 2**). Cluster I was further sub-grouped into gene functions related to protein catabolism and metabolism and thus, might influence the rates of protein turnover. Genes in cluster 8 are up-regulated in *idd4* after salt-stress but down-regulated in WT. These genes are categorized in functional GO terms for the response to jasmonic and salicylic acid and contribute to immune reactions and defense adaptation. Interestingly, DEGs in cluster 3 revealed gene functions that can be grouped in GO terms for response to abiotic and ABA stimulus, salt-stress, and hyperosmotic response, as well as response to water deprivation. These DEGs are up-regulated in *idd4* after salt-exposure compared to WT, suggesting that IDD4 is genetically linked to the repression of abiotic/salt-response factors. Notably, genes in cluster 3 are dedicated to response to auxin-stimulus and might contribute to the processes that promote growth and development following salt-stress. The comparative transcriptomic approach revealed the up-regulation of genes in *idd4* that are involved in the regulation of salt-stress adaptation, thereby suggesting *IDD4* as a repressor of salt-stress response.

### Primary target genes of IDD4 contribute to the salt-stress response

**Figure 7:**
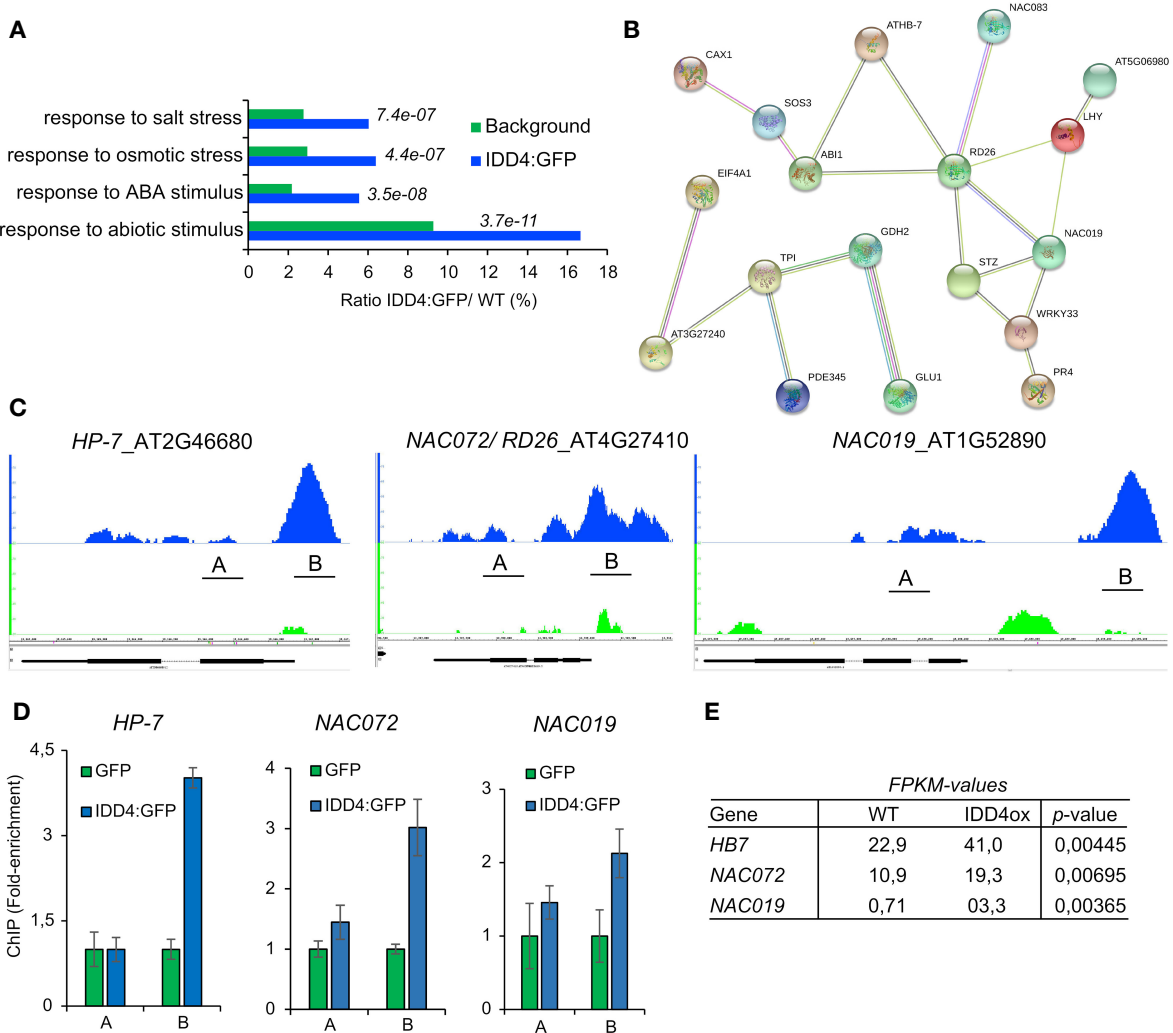
Genome-wide identification of IDD4 chromatin-bound regions involved in abiotic stress response **(A)** Gene ontology annotations of significant ChIP-SEQ targets. **(B)** Protein interaction networks derived from the IDD4 ChIP-SEQ targets grouped in the GO response to salt-stress. Network was generated by the use of STRING (version 10.0). Minimum required interaction score is defined as medium confidence 0.5, Meaning of network edges “evidence”. **(C, D)** Binding profiles of IDD4 to the *HP7*, *NAC072* and *NAC019* loci. ChIP-SEQ profiles **(C)** and ChIP-qPCR evaluations **(D)** are depicted for each locus. The TAIR annotations of the genomic loci are shown at the bottom of each panel. The genomic locus indicated above the scale represents forward (+) orientation, while the one below represents reverse orientation. In each case, the enrichment was found to be in the upstream region of the respective genomic locus. **(E)** Transcript amounts of *HP7*, *NAC072* and *NAC019* are increased in IDD4 over-expressor lines compared to WT.

To unravel the molecular network of IDD4 that coordinates salt-stress responses, we pursued to identify direct IDD4 target genes. We thus analyzed the genomic regions which are *in vivo* targeted by IDD4. By exploiting chromatin-immune precipitation studies followed by deep-sequencing on the Illumina-high sequencing platform (ChIP-SEQ) (Völz et al., 2019b), we identified primary downstream targets of IDD4 that were enriched in GO terms for ‘response to salt and osmotic stress’, ‘ABA and abiotic stimulus’, thereby demonstrating salinity-associated target genes of IDD4 (**Figures 7A, B**).

Genes that were categorized with the GO term ‘salt-stress’ formed a functional network, emphasizing that these factors are interconnected with each other in the process of salt-stress regulation (**Supplementary Data 3**). Among these, ARABIDOPSIS THALIANA HOMEOBOX 7 (HB-7) was shown to fine-tune processes associated with growth and response to water stress (Re et al., 2014). Ectopic expression of HB-7 induced tolerance to drought, salinity, and oxidative stress (Pruthvi et al., 2014). The ARABIDOPSIS NAC DOMAIN CONTAINING PROTEIN (NAC) 072/RD26, NAC019, NAC083 (**Supplementary Figure 4A**) are important stress-response integrators that exert different functions during the salt-stress response by signal integration of growth hormones in favor of abiotic stress compensation (Takasaki et al., 2015; Wang et al., 2015). The IDD4 binding profiles of HB-7, NAC072/RD26, and NAC019 generated by ChIP-SEQ were confirmed by ChiP-qPCR (**Figures 7C, D**), indicating the association of IDD4 to the 5’ upstream regions of the respective gene loci. Genome-wide expression data of IDD4 overexpressor lines (Völz et al., 2019b) unveiled higher transcript levels of these factors (**Figure 7E**) compared to WT, suggesting that IDD4 acts as a transcriptional activator. Furthermore, we also found IDD4 to be associated with the promoter regions of several well-known salt-stress regulators, such as two members of the salt-overly-sensitive (SOS) pathway, which participate in ion-homeostasis regulation. *SOS3* and *SOS3-INTERACTING PROTEIN 2* (*SIP2*) encode a sensor of cytosolic Ca^2+^ that is essential for K^+^ nutrition, K^+^/Na^+^ selectivity, and salt tolerance (Ishitani et al., 2000; Li et al., 2013; Van Oosten et al., 2013) (**Supplementary Figures 4B, C**). IDD4 was also found to be associated with the promoter regions of the genes for the protein phosphatase 2C ABA-insensitive 1 (*ABI1*) (**Supplementary Figure 4E**), which is a key component of the ABA core signaling complex, and the transcription factor *WRKY33* (**Supplementary Figure 4D**), which is also involved in ABA signal transduction of salinity-stress (Jiang and Deyholos, 2009). The association of IDD4 to these target genes indicates its direct influence on factors shaping the salt-stress response of *Arabidopsis* (**Figure 7**; **Supplementary Figure 4**).

## Discussion

**Figure 8:**
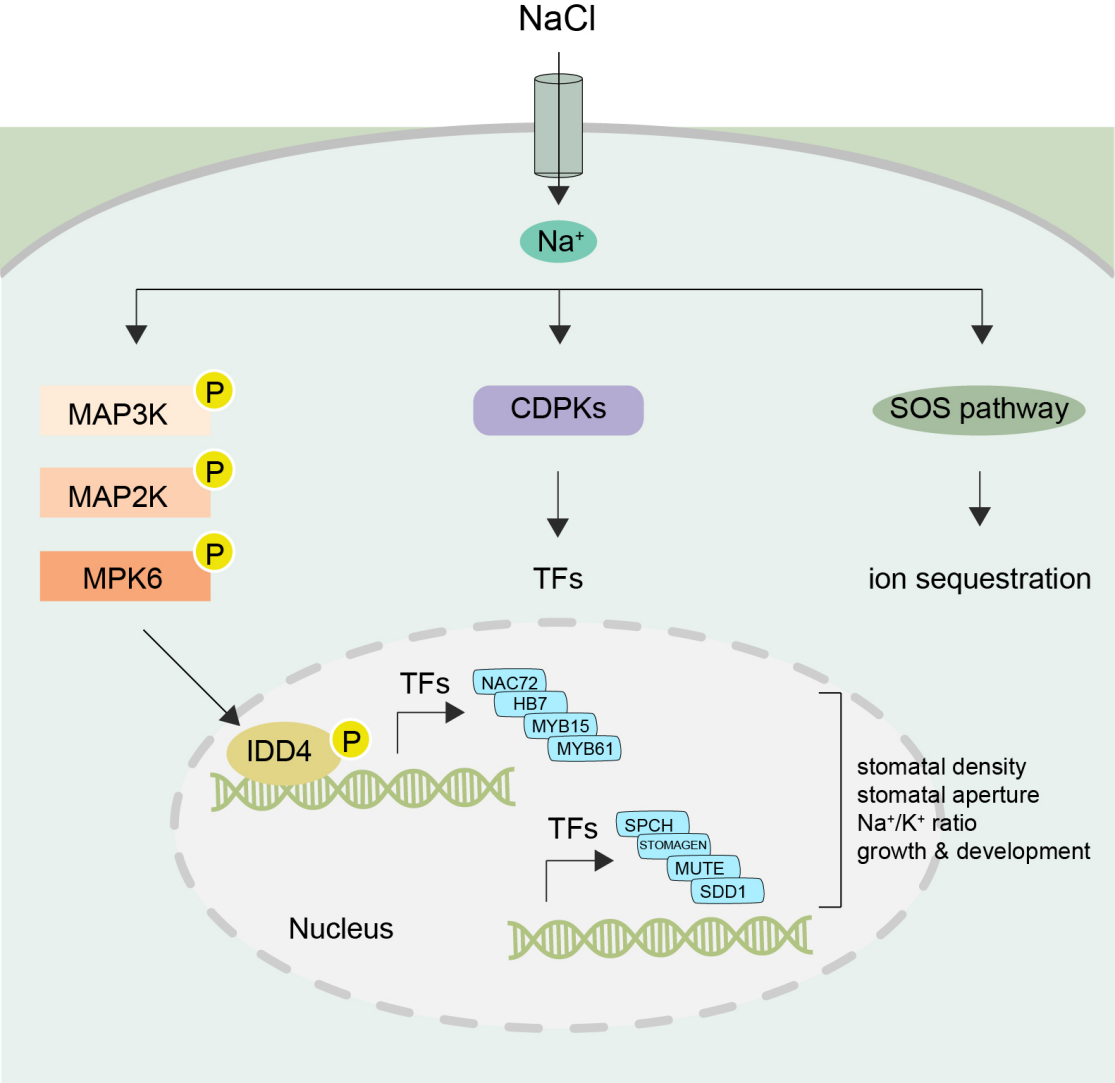
Working model of IDD4 activation and signaling under salt-stress Upon perception of NaCl signals the MAPK cascade becomes activated resulting in the activation of IDD4 (Indeterminate-domain 4) via MPK6 (mitogen-activated protein kinase 6). The phosphorylated IDD4 binds to the promoter regions of its target genes, e.g. transcription factors (TFs) controlling the genes involved in salt response or stomatal patterning, and thereby regulating the downstream stress responsive target genes. Thus, the MPK6-IDD4 module regulates the salt-stress response under unfavorable saline conditions. SOS, salt overly sensitive; CDPK, Ca^2+^ dependent protein kinase; MAP3K-MAP2K-MAPK (mitogen-activated protein kinase) cascade. SPCH, Speechless; SDD1, Stomatal density and distribution 1; NAC, (NAM, ATAF1/2, and CUC2); HB7, Homeobox7; MYB, myeloblastosis viral oncogene homolog.

The IDD family of transcription factors regulate several biological processes in plants (Kumar et al., 2019) and several phosphoproteomic studies have identified IDD transcription factors as targets for phosphorylation (Völz et al., 2019c). We previously showed that *IDD4* regulates plant immune responses and its activity is controlled by phosphorylation through MPK6 (Völz et al., 2019b). Here we show that IDD4 is involved in regulating salt tolerance and growth. We propose a gene regulatory network model, whereby MPK6 phosphorylates IDD4 to activate the expression of a cascade of downstream transcription factors, including HB7, NAC19 and NAC72 to adjust growth and development of *Arabidopsis* under salt-stress conditions (**Figure 8**).

### *In-vivo* phosphorylation of *IDD4*

A multitude of phosphoproteomic studies suggest conserved phosphosites within the members of the IDD-family (Völz et al., 2019c). MPK6 was shown to be phosphorylated and thereby activated following salt-stress (Ichimura et al., 2000). In this context, we previously showed that activated-MPK6 phosphorylates IDD4 (Völz et al., 2019b). A major phosphosite found in all IDD-members is a serine at position 73 in IDD4 upstream of ZF1 that is targeted by MPK6 in response to salinity stress (Wang et al., 2020). By using a phosphospecific IDD4-S-73 antibody, we confirmed that salt-stress triggers the phosphorylation of IDD4, These findings, together with the corresponding enhanced salt-resistant phenotype of the *mpk6-2* and *idd4* mutant are coherent with evidence that phosphorylation of IDD4 regulates its DNA-binding ability and expression of downstream target genes (Völz et al., 2019b).

### MPK6 negatively regulates salt-stress adaptation

MPK6 is known as a negative regulator of salt-stress (Alzwiy and Morris, 2007; Han et al., 2014; Liu et al., 2015), playing a crucial role in the Na^+^ uptake and Ca^+^ homeostasis of *Arabidopsis* roots (Han et al., 2014). Our salt-stress phenotyping of *mpk6* confirms that MPK6 acts as a negative regulator of salinity stress (**Figure 6**; **Supplementary Figure 4**). The kinase activity of MPK6 is rapidly triggered in a time- and dose-dependent manner by salt-stress, and this activation is induced mainly by phosphorylation of the TEY motif in the activation loop of the MAPKs (Ichimura et al., 2000; Yu et al., 2010; Liu et al., 2015). MAPKs regulate their substrates through the phosphorylation of conserved Ser/Thr residues adjacent to proline residues (S/T-P). IDD4 harbors a conserved MAPK docking site between ZF1 and ZF2 and has been shown to interact predominantly with MPK6, which phosphorylates IDD4 on S-73 as well as on Thr-130 (Völz et al., 2019b). The importance of MPK6-mediated IDD4 phosphorylation in response to salt stress was evidenced by the enhanced and reduced salt tolerance phenotypes of *IDD4-AA* (phospho-dead) and *IDD4-DD* (phospho-mimic) transgenic lines respectively.

### IDD4 regulates Na^+^/K^+^ ratios under salinity

Salinity causes an increase in the concentrations of Na^+ ^and Cl^–^ leading to cytotoxicity, thereby inhibiting plant growth and development (Isayenkov and Maathuis, 2019). Increases in Na^+^ lead to decreased K^+^ concentrations in plant cells, resulting in detrimental Na^+^/K^+^ ratios. Plants can counteract the harmful effects of ionic stress, caused by hyper-accumulation of Na^+^, by increasing K^+^ uptake. The main contributors to root K^+^ uptake are AKT1 and HAK5, and HAK5 has been shown to be induced upon K^+^ starvation (Gierth et al., 2005). When exposed to salinity stress, several *Arabidopsis* salt tolerant accessions, showed no change in Na^+^ but higher K^+ ^and lower Na^+^/K^+^ ratios, along with the up-regulation of *HAK5*, *CHX17* and *KUP1* (Sun et al., 2015). The *idd4* transcriptome showed increased expression of *HAK5*, which could contribute to the enhanced K^+^ content and the reduced Na^+^/K^+^ ratio (**Figure 3**), making *idd4* plants more salt-stress tolerant. Binding studies of IDD4 revealed primary downstream targets related to salt-stress adaptation by the integration of growth hormone pathways. These primary targets predominantly belong to the group of NAC TFs. Interestingly, the genomic loci encoding for components of the cytosolic Ca^2+^ sensor SOS3 and SIP2, essential for K^+^ nutrition, K^+^/Na^+^ selectivity, and salt tolerance, were also found to be targets of IDD4.

### Regulation of stomatal density and closure by IDD4

Plants utilize several mechanisms to overcome salinity stress, including the rapid stomatal closure upon salt exposure (Munns and Tester, 2008). Several studies show that *MPK6* induces stomatal closure, and can do so independently of ABA signaling (Montillet et al., 2013; Su et al., 2017). We found the two key transcription factors *MYB15 and MYB61* to be differentially regulated in *idd4* (**Supplementary Data 1**) and *IDD4ox* (Völz et al., 2019b) respectively. *MYB15* participates in the regulation of stomatal closure and shows altered drought and salinity tolerance in Arabidopsis (Ding et al., 2009), while *MYB61* regulates stomatal pores in an ABA-independent manner (Liang et al., 2005). The altered stomatal closure of *idd4* and *IDD4* overexpressing plants (**Figure 2**) could be due to the regulation of the *MYB15* and *MYB61* genes by IDD4 (**Supplementary Data 1**) (Völz et al., 2019b), which are involved in the regulation of stomatal movement (Cominelli et al., 2010). The overexpression of *MYB15* seen in *idd4* mutants might lead to enhanced stomatal closure in response to salt, making these plants more stress tolerant (Ding et al., 2009). Interestingly, the larger stomatal pore size of *IDD4ox* plants might be linked to the downregulation of *MYB61*, mutants of which also result in larger stomatal pores (Liang et al., 2005). In line with this idea, we recently reported that plants expressing a dominant-negative version of *IDD4* (*idd4SRDX*) are affected in the stomatal opening/closing mechanism (Völz et al., 2019a), and stomata of *idd4SRDX* lines show generally reduced stomatal aperture, as observed in the *idd4* mutant plants. Furthermore, the expression of *idd4SRDX* compromises the stomatal reopening after infection by the pathogen *Pseudomonas syringae DC3000*. In our study *of idd4SRDX* lines, we identified several direct IDD4 target genes involved in stomatal movement and patterning, indicating that IDD4 acts as an upstream regulator of *STOMAGEN* and *SPEECHLESS*, two key-factors in stomatal development. In addition, *STOMATAL DENSITY AND DISTRIBUTION 1 (SDD1)* and the basic helix-loop-helix protein *MUTE*, which control meristemoid differentiation during stomatal development, were also identified as targets of IDD4 (O'Malley et al., 2016; Völz et al., 2019a). The genetic interaction between the above mentioned genes and IDD4 is in accordance with the observed changes in stomatal density and pore opening in *IDD4* transgenic lines. Hence, our data further emphasize IDD4 as upstream regulator of pivotal factors controlling stomatal patterning and aperture.

### Coordination of salt-stress responses by IDD4 and DELLA proteins

Our previous study showed that *IDD4* regulates both the growth and immune response of *Arabidopsis*, suggesting that the trade-off model between plant growth and defense may be too simplistic (Völz et al., 2019b). In accordance with the transcriptome analysis, which suggested that IDD4 might also be involved in regulating salt-stress response genes, *IDD4* knock out and overexpression lines show alterations in salinity tolerance. While *IDD4* loss-of-function resulted in better seed germination as well as shoot and root growth under salt-stress conditions, *IDD4* overexpression increased the salt sensitivity during germination and vegetative growth (**Figure 1**). An important mediator of biotic and abiotic stress signals including salinity is the transcriptional regulator DELLA. In line with this concept, quadruple DELLA mutant plants exhibit enhanced salt tolerance (Achard et al., 2006). Since it was previously shown that IDD4 interacts with DELLA/RGA to regulate immune response genes (Yoshida et al., 2014), it is likely that the cooperative action of IDD4 and DELLAs might also function in a similar fashion to regulate salt tolerance in plants.

Our work shows that the function of *IDD4* regulates plant growth and development under abiotic stress conditions, such as salt stress. Our work proposes that *MPK6* and *IDD4* are activated upon salt stress sensing and that MPK6 is a key regulator of IDD4 activation. Phosphorylated IDD4 binds to the promoters of its downstream target genes to regulate their expression. A number of direct IDD4 targets encode key factors in determining stomatal density and behavior. Moreover, IDD4 targeted transcription factors, such as NAC72 and HB-7, themselves regulate a number of salt-stress response genes (**Figure 8**). In this way, the MPK6-IDD4 regulated signaling module coordinates growth and development with stress responses to optimally adapt plants to salt-stress conditions.

## Data availability statement

The datasets presented in this study can be found in online repositories. The names of the repository/repositories and accession number(s) can be found below: GEO accession number: GSE242075.

## Author contributions

AR: Conceptualization, Investigation, Methodology, Writing – original draft. RV: Conceptualization, Investigation, Methodology, Writing – original draft. AS: Investigation. KM: Data curation, Software. S-KK: Investigation, Methodology. NR: Methodology. KA: Investigation. LA: Resources. MB: Methodology. IB: Resources. HH: Conceptualization, Formal Analysis, Project administration, Supervision, Visualization, Writing – review & editing.

## Supplementary material

Supplementary Figure 1 Phenotype of *Arabidopsis idd4* mutant plants under non-salt conditions.

Supplementary Figure 2 Binding of IDD4-pS73 antibody to phosphorylated IDD4 in *IDD4ox.*

Supplementary Figure 3 Phenotype of *Arabidopsis IDD4-AA and IDD4-DD* mutant lines under non-salt conditions.

Supplementary Figure 4 Binding profiles of IDD4 to salt-stress responsive genes.
