# Supplementary material for: Salinity stress-induced phosphorylation of INDETERMINATE-DOMAIN 4 (IDD4) by MPK6 regulates plant growth adaptation in Arabidopsis
Source: Front Plant Sci. 2023 Oct 10;14:1265687. doi: 10.3389/fpls.2023.1265687 (PMC10595144; doi:10.3389/fpls.2023.1265687)
Supplement: Supplementary Figure 1 — Phenotype of Arabidopsis idd4 mutant plants under non-salt conditions. [file DataSheet_1.pdf]

Salinity stress-induced phosphorylation of INDETERMINATE-DOMAIN 4 (IDD4) by MPK6 regulates plant growth adaptation in *Arabidopsis*

Anamika A. Rawat[1*], Ronny Völz[1], Arsheed Sheikh[1], Kiruthiga G. Mariappan[1], Soon-Kap Kim[1], Naganand Rayapuram[1], Khairiah M. Alwutayd[2], Louai K. Alidrissi[1], Moussa Benhamed[3], Ikram Blilou[1], Heribert Hirt[1,4*]

<u>SUPPLEMENTARY FIGURES</u>

Supplemental Figure 1

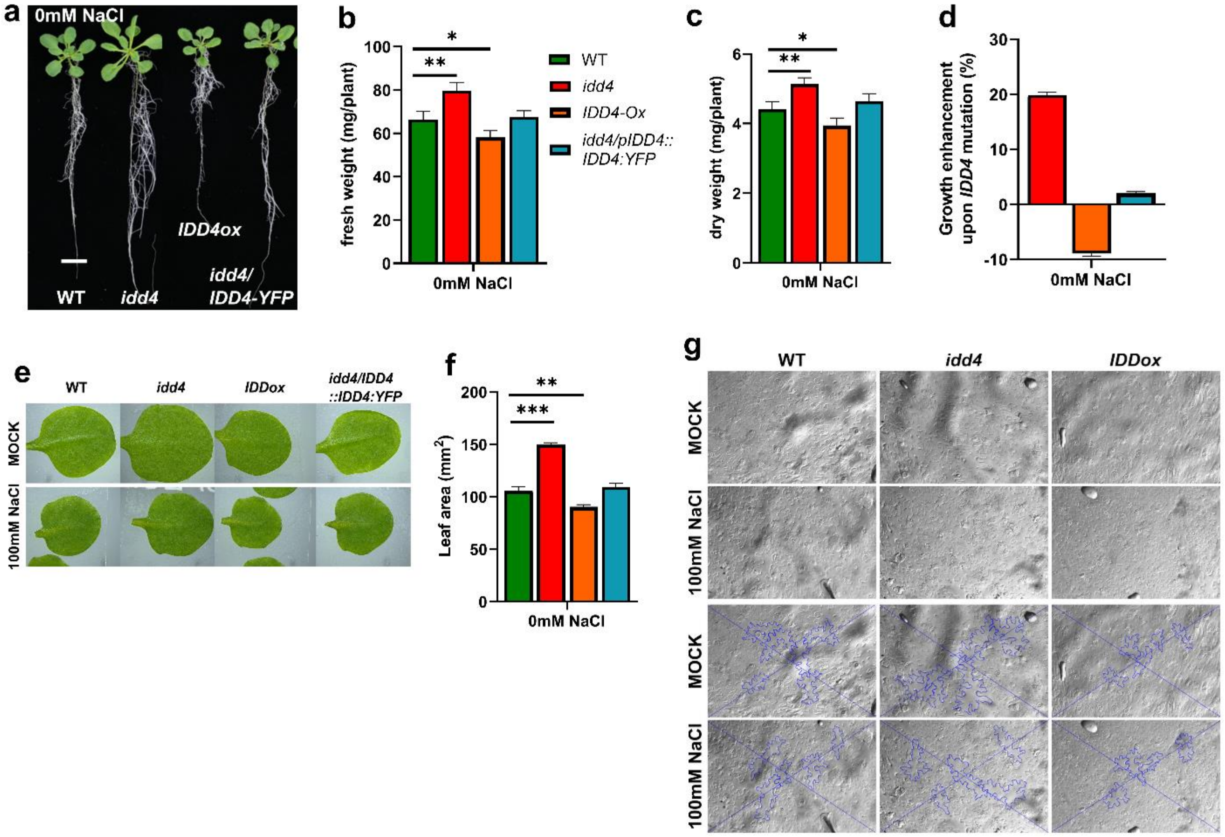

Supplemental Figure 1. The phenotype of *Arabidopsis idd4* mutant plants under non-salt conditions

a Morphology of WT, *idd4*, *IDD4ox* and complementation lines 16d post non-salt treatment. Scale bar = 1 cm. **b** The fresh weight of plants after 16d of 0mM salt treatment. Values represent average ± SE ($n \geq 25$). **c** The dry weight of plants after 16d of 0mM salt treatment. Values represent average ± SE ($n \geq 25$). **d** The effect of *IDD4* on the growth of *Arabidopsis* under non-salt stress condition. **e** A leaf of WT, *idd4,* IDD4ox and complementation line from 16d post ± 100mM salt treated plants. Scale bar = 5 mm. **f** The leaf area from (a). **g** Representative image of epidermal peel showing pavement cells in WT, *idd4,* and *IDD4ox*.

Each bar in the graphs represents an average of three biological replicates ± SE; $n \geq 18$. * $p \leq 0.05$, ** $p \leq 0.01$, *** $p \leq 0.001$ (Student's *t*-test).

Supplemental Figure 2

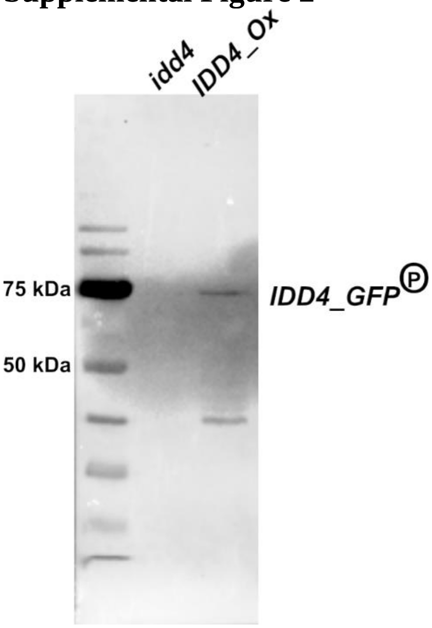

Supplemental Figure 2. Binding of IDD4-pS73 antibody to phosphorylated IDD4 in *IDD4ox* after 150mM of NaCl treatment. For *idd4* the total protein extract was used while for *IDD4_Ox* the IDD4 protein was pulled down using GFP beads.

Supplemental Figure 3

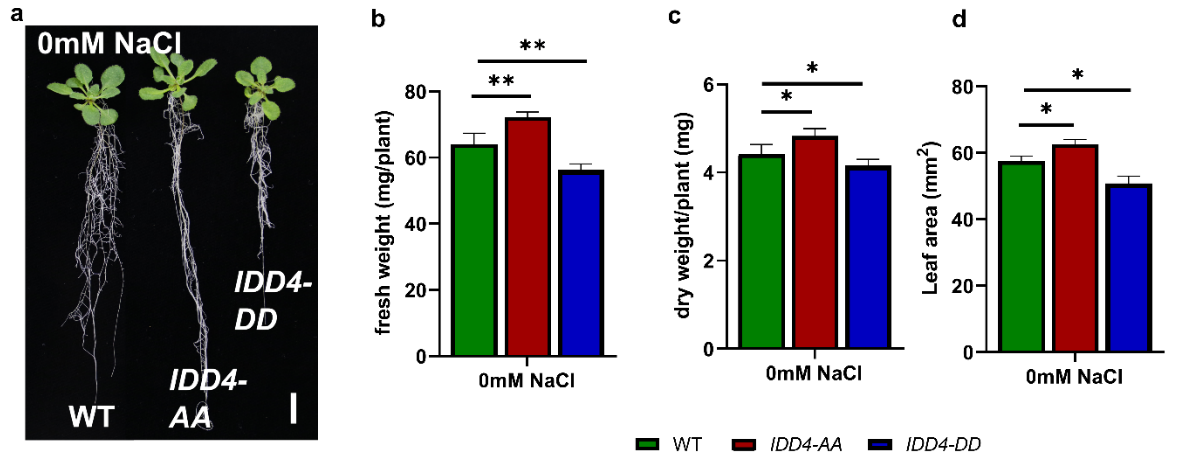

Supplemental Figure 3. Phenotype of *Arabidopsis IDD4-AA and IDD4-DD* mutant lines under non-salt conditions.

a Morphology of WT, *IDD4-AA and IDD4-DD* lines after 16d of non-salt-stress. Scale bar = 1 cm. **b** The fresh weight of plants after 16d of 0mM salt treatment. Values represent average ± SE ($n \geq 25$). **c** The dry weight of plants after 16d of 0mM salt treatment. Values represent ± SE ($n \geq 25$). **d** Leaf area of WT, *IDD4-AA and IDD4-DD* mutant lines from (a). Values represent average ± SE, ($n \geq 25$).

Each bar in the graphs represents an average of three biological replicates ± SE; * $p \leq 0.05$, ** $p \leq 0.01$, *** $p \leq 0.001$ (Student's *t* test).

Supplemental Figure 4

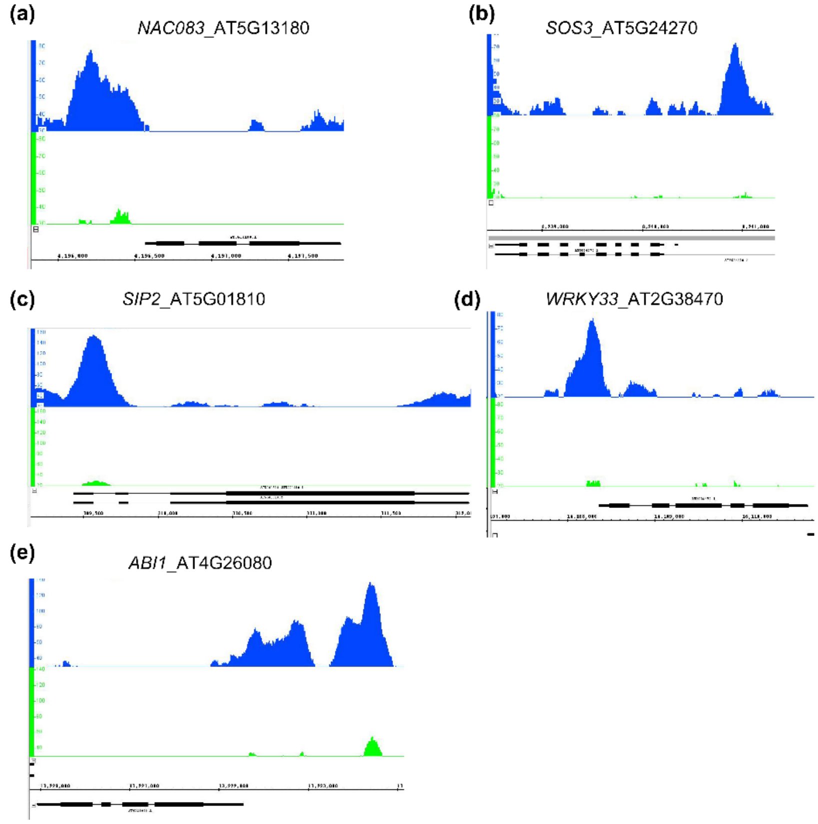

Supplemental Figure 4. Binding profiles of IDD4 to salt-stress responsive genes

a-e ChIP-SEQ based-binding proles of IDD4 to the NAC083, SOS3, SIP2, WRKY33 and ABI1 loci.

The TAIR annotations of the genomic loci are shown at the bottom of each panel.

The genomic locus indicated above the scale represents forward (+) orientation, while the one below represents reverse orientation. In each case, the enrichment was found to be in the upstream region of the respective genomic locus.
